# High expression of soluble CD155 in estrogen receptor-negative breast cancer

**DOI:** 10.1007/s12282-019-00999-8

**Published:** 2019-08-01

**Authors:** Akiko Iguchi-Manaka, Genki Okumura, Emika Ichioka, Hiroko Kiyomatsu, Tatsuhiko Ikeda, Hiroko Bando, Akira Shibuya, Kazuko Shibuya

**Affiliations:** 1 Department of Breast and Endocrine Surgery, Faculty of Medicine, University of Tsukuba, Tsukuba, 305-8575 Japan; 2 Department of Immunology, Faculty of Medicine, University of Tsukuba, Tsukuba, 305-8575 Japan; 3 Life Science Center for Survival Dynamics, Tsukuba Advanced Research Alliance (TARA), University of Tsukuba, Tsukuba, 305-8575 Japan; 4 Department of Breast and Endocrine Surgery, University of Tsukuba Hospital, Tsukuba, 305-8576 Japan

**Keywords:** Soluble CD155, DNAM-1, ER-negative, Ki-67-high

## Abstract

**Background:**

The poliovirus receptor (CD155) is expressed ubiquitously at low levels on both hematopoietic and nonhematopoietic cells, but its expression is upregulated in various tumor cells. An activating receptor DNAM-1 expressed on cytotoxic T lymphocytes (CTLs) and natural killer (NK) cells binds to CD155 and mediates the cytotoxic activity of CTLs and NK cells against tumors. Unlike mouse tissues, human tissues express a soluble form of CD155 (sCD155), which is a splicing isoform of CD155 lacking the transmembrane region. We previously reported that the serum levels of sCD155 were higher in lung, gastrointestinal, breast, and gynecologic cancer patients than in healthy donors. Here, we focus on breast cancer patients.

**Methods:**

To analyze the association between serum level of sCD155 and clinicopathological parameters of breast cancer, we quantified sCD155 in the sera of 153 breast cancer patients by sandwich ELISA.

**Results:**

sCD155 levels in the sera of breast cancer patients were positively correlated with patient age, disease stage, and invasive tumor size. Moreover, they were higher in patients with estrogen receptor (ER)-negative cancers than in those with ER-positive tumors, and higher in those with Ki-67-high cancers than in those with Ki-67-low cancers.

**Conclusions:**

The serum level of sCD155 is correlated with high risk factors in breast cancer.

## Introduction

The interactions between the immune system and cancer are complicated, because the immune system plays dual roles of cancer suppression and promotion [1, 2]. Cytotoxic T lymphocytes (CTLs) and natural killer (NK) cells play key roles in tumor suppression by mediating tumor recognition and activation through their antigen receptors and a variety of adhesion and costimulatory molecules [3, 4]. Interactions between cell surface receptors on CTLs and NK cells and their ligands expressed on tumor cells induce cytotoxic activity against tumor cells [5].

DNAM-1 (CD226) is a member of the immunoglobulin superfamily and is expressed on NK cells, T cells, monocytes, macrophages, and platelets [6, 7]. Its ligands in humans and mice are CD155 (also called poliovirus receptor [PVR]) and its family member CD112 (also called PVR-related family 2 [PRR-2] or nectin-2) [8–10]. Human CD155 and CD112 are broadly distributed on epithelial and endothelial cells as well as hematopoietic cells and are overexpressed on various tumors [11–15]. Interactions between DNAM-1 on CTLs and NK cells and CD155 and CD112 on tumor cells augment the cytotoxicity of these cells against tumors [8, 9]. Chemical carcinogen-induced tumor models using DNAM-1-deficient mice have shown that DNAM-1 plays an important role in immune surveillance against CD155-expressing tumors [16].

Unlike mouse tissues, which express membrane-bound CD155 (mCD155), human tissues also express soluble CD155 (sCD155) encoded by splicing isoforms of CD155 (CD155β and CD155γ) that lack exon 6 encoding the transmembrane region [17, 18]. We previously reported that serum levels of sCD155 were higher in patients with various cancer types (lung, gastrointestinal, breast, and gynecologic) than in healthy donors. Moreover, they were higher in patients with advanced-stage gastric cancer compared with an early stage of the cancer [19].

In recent years, diagnosis and therapy for breast cancer have made remarkable progress on the basis of biological research. In 2000, intrinsic subtypes of breast cancer were identified according to gene-expression patterns [20]. Breast cancers were then classified into five subtypes based on the expression levels of estrogen receptor (ER), progesterone receptor (PgR), human epidermal growth factor receptor 2 (HER2), and Ki-67 in tumor cells, as determined by immunohistochemical and gene-expression analyses (St. Gallen International Expert Consensus on the Primary Therapy of Early Breast Cancer 2011) [21]. These subtypes exhibit different epidemiologies, natural histories, and responses to therapies [21]. Therefore, characterization of the intrinsic subtypes of breast cancer is important for the development of appropriate therapies.

Here, we analyzed the association between serum levels of sCD155 and the clinicohistopathological features of a cohort of 153 breast cancer patients.

## Materials and methods

### Samples

Serum samples were obtained from patients admitted to the University of Tsukuba Hospital, Japan, for primary treatment of breast cancer. Cases of multiple, bilateral breast cancers or multiple primary cancers were excluded from this study. Patients who received neoadjuvant therapy were also excluded. Written informed consent was obtained from all individual participants included in the study. The study was approved by the ethics committee of the University of Tsukuba (approval number, 531-5). Disease stage was classified according to The Union for International Cancer Control (UICC) TNM Classification of Malignant Tumors. Surgically treated cases were classified pathologically, and other cases were classified clinically. ER and PgR statuses were evaluated by immunostaining. Human epidermal growth factor receptor 2 (HER2) status was evaluated by immunostaining, and in cases with a score of 2+, it was evaluated by fluorescence in situ hybridization. Cases of ductal carcinoma in situ were not included in the analyses of nuclear grade; ER, PgR, and HER2 statuses; or Ki-67 index.

### ELISA for human soluble CD155

In brief, Nunc-Immuno Plates (Thermo Fisher Scientific, MA) were coated with anti-CD155 antibody (D171 [Thermo Fisher Scientific, MA], 0.2 µg/mL in carbonate-bicarbonate buffer, pH 9.3–9.9 [Sigma-Aldrich, MO], 100 µL/well) for capture for 1 h at room temperature (RT), washed three times with washing buffer (PBS containing 0.05% TWEEN 20 [Sigma-Aldrich, MO]), and treated with blocking buffer (washing buffer containing 0.5% bovine serum albumin fraction V [Roche, Switzerland]) for 1 h at RT. After the plates had been washed again, human chimeric protein consisting of the extracellular portion of CD155β fused with Flag peptide at the C-terminus (CD155β-FLAG) (as a standard; provided by Chugai Pharmaceutical Co., Ltd., Japan) and serum samples of patients (1:100 in blocking buffer) or control pooled sera (Biopredic International, France, Human True A serum, pool of donors, 1:100 in blocking buffer) were plated at 100 µL/well, incubated for 1 h at RT, washed, and then incubated with anti-Cynomolgus monkey PVR antibody (Sino Biological, China, 0.2 µg/mL in blocking buffer, 100 µL/well). This was followed by treatment with biotinylated monoclonal anti-rabbit IgG (γ-chain specific) antibody (Sigma-Aldrich, MO, 1:5000 in blocking buffer, 100 µL/well). The plates were then incubated with Streptavidin-Poly HRP 80 (SDT, 1:5000 in blocking buffer, 100 µL/well). This was followed by reaction with 100 µL of TMB One Component HRP Microwell Substrate (BioFX Laboratories, MD) for 5 min at RT. The reactions were stopped with 50 µL of 0.5 mol/L sulfuric acid, and the absorbance of each well at a wavelength of 450 nm was measured with a Spectra Max M2e reader (Molecular Devices, CA). All values were determined in triplicate.

### CD155 expression analysis using The Cancer Genome Atlas open access data

RNA expression data of CD155α and CD155γ in normal and cancerous breast tissues were extracted from The Cancer Genome Atlas (TCGA) open access data in January 2014 (https://tcga-data.nci.nih.gov/docs/publications/tcga/). For both CD155α and CD155γ, expression ratios of cancer tissue versus normal tissue were calculated using the following formula: CD155α or γ expression ratio = read counts of CD155α or γ-specific junction (CD155 Exon 6b-7) in cancer tissue/read counts of CD155α γ-specific junctions in paired normal tissue.

### Statistics

Statistical analyses were performed using Spearman rank-order correlation and the two-tailed Mann–Whitney *U* test. All analyses were performed with GraphPad Prism (GraphPad Software, CA). *P* values less than 0.05 were considered to be statistically significant.

## Results

### Correlation between clinicopathological parameters and serum levels of sCD155 in breast cancer patients

**Fig. 1:**
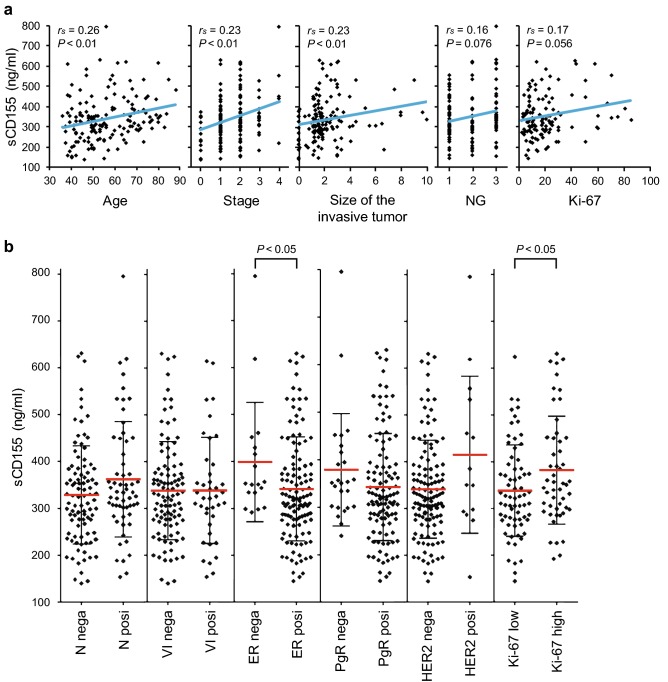
Correlation between clinicopathological parameters and serum levels of sCD155 in breast cancer patients. **a** Scatter plot, regression line, and correlation coefficient between clinicopathological parameters and serum levels of sCD155. **b** Comparisons of serum levels of sCD155 in patients grouped by the indicated pathological parameters. *r*_*s*_ Spearman rank-order correlation, *NG* nuclear grade, *N* lymph node metastasis, *nega* negative, *posi* positive, *VI* vascular invasion, *ER* estrogen receptor, *PgR* progesterone receptor, *HER2* human epidermal growth factor receptor 2

To analyze the association between serum level of sCD155 and clinicopathological parameters of breast cancer, we quantified sCD155 in the sera of 153 breast cancer patients before primary treatment by sandwich ELISA. Of these patients, 149 were treated surgically and their pathological parameters were determined by pathohistological analysis of the surgical specimen. The other 4 patients, who were all at stage 4, were treated without surgery; their clinicopathological parameters were determined by imaging diagnosis and core needle biopsy of the primary tumor. sCD155 levels in the sera of the 153 breast cancer patients were significantly positively correlated with patient age, disease stage, and tumor size (Table 1, Fig. 1a). Furthermore, the levels were significantly higher in patients with ER-negative versus ER-positive cancers and in patients with high versus low Ki-67 cancers (Table 1, Fig. 1b). As a reference, the median level of sCD155 in the purchased pooled sera of 26 healthy donors (Biopredic International) was 289.96 ng/mL, which was lower than the median of ER-positive and low Ki-67 cancers. These results suggest that serum levels of sCD155 are associated with the clinicopathological characteristics of breast cancer patients.

**Table 1:** Clinicopathological parameters and serum levels of sCD155 in breast cancer patients

Clinicopathological parameter	Number (%)	sCD155 (ng/mL)			
		Mean	Median	Range	Significance
Age (years)					
36–39	12 (7.8)	339.6	338.5	162.5–610.4	*r*_*s*_ = 0.26
40–49	44 (28.8)	309.6	311.0	139.8–586.1	*P *< 0.01*
50–59	41 (26.8)	332.5	307.4	144.8–795.3	
60–88	56 (36.6)	373.3	356.7	192.6–623.9	
Treatment					
Surgery	149				
Other	4				
Histological type					
Ductal carcinoma in situ	16 (10.5)	276.2	280.7	139.8–376.0	
Invasive carcinoma of NST	118 (77.1)	354.1	330	144.8–795.3	
Invasive lobular carcinoma	14 (9.2)	318.6	325	188.7–482.9	
Invasive micropapillary carcinoma	2 (1.3)				
Apocrine carcinoma	2 (1.3)				
Mucinous carcinoma	1 (0.7)				
Stage					
0	16 (10.5)	276.2	280.7	139.8–376.0	*r*_*s*_ = 0.23
1	56 (36.6)	328.9	314.4	144.8–630.6	*P *< 0.01*
2	59 (38.6)	355	325.7	153.7–623.9	
3–4	22 (14.4)	384.2	353.9	188.7–795.3	
Size of invasive tumor (cm)					
0.0–2.0	96 (62.7)	320.1	310.9	139.8–630.6	*r*_*s*_ = 0.23
2.1–5.0	41 (26.8)	367.7	334.3	153.7–623.9	*P *< 0.01*
≥ 5.1	11 (7.2)	360.1	356.9	188.7–532.2	
Unknown	5 (3.3)				
Lymph node metastasis					
Negative	94 (61.4)	328.5	321.3	139.8–630.6	*P *= 0.14^a^
Positive	59 (38.6)	362	334.3	153.7–795.3	
Vascular invasion					
Negative	102 (66.7)	337.9	328.2	139.8–630.6	*P *= 0.91^a^
Positive	40 (26.1)	338.4	323.8	153.7–614.6	
Unknown	11 (7.2)				
Nuclear grade (except for DCIS)					
1	59 (43.1)	333.6	310.8	144.8–556.6	*r*_*s*_= 0.17
2	31 (22.6)	332.1	306	161.4–623.9	*P *= 0.055
3	36 (26.3)	390.3	352.3	153.7–795.3	
Unknown	11 (8.0)				
ER status (except for DCIS)					
Negative	18 (13.1)	398.7	352.4	291.8–795.3	*P *< 0.05*^a^
Positive	119 (86.9)	341.5	321.9	144.8–630.6	
PgR status (except for DCIS)					
Negative	25 (18.2)	378.7	351.3	239.7–795.3	*P *= 0.14^a^
Positive	112 (81.8)	342.4	322.6	144.8–630.6	
HER2 status (except for DCIS)					
Negative	123 (89.8)	341.5	325.7	144.8–630.6	*P *= 0.13^a^
Positive	14 (10.2)	415	368	153.7–795.3	
Ki-67 (except for DCIS)					
Low (0–19%)	69 (50.4)	338.1	324.7	144.8–623.9	*P *< 0.05*^a^
High (20–100%)	51 (37.2)	382	354	192.6–630.6	
Unknown	17 (12.4)				

*sCD155* soluble CD155, *r*_*s*_ Spearman rank-order correlation, *NST* no special type, *DCIS* ductal carcinoma in situ, *ER* estrogen receptor, *PgR* progesterone receptor, *HER2* human epidermal growth factor receptor 2

*Significant *P* values

^a^Mann–Whitney *U* test

### Expression analysis of CD155α and CD155γ mRNA in breast cancer

**Fig. 2:**
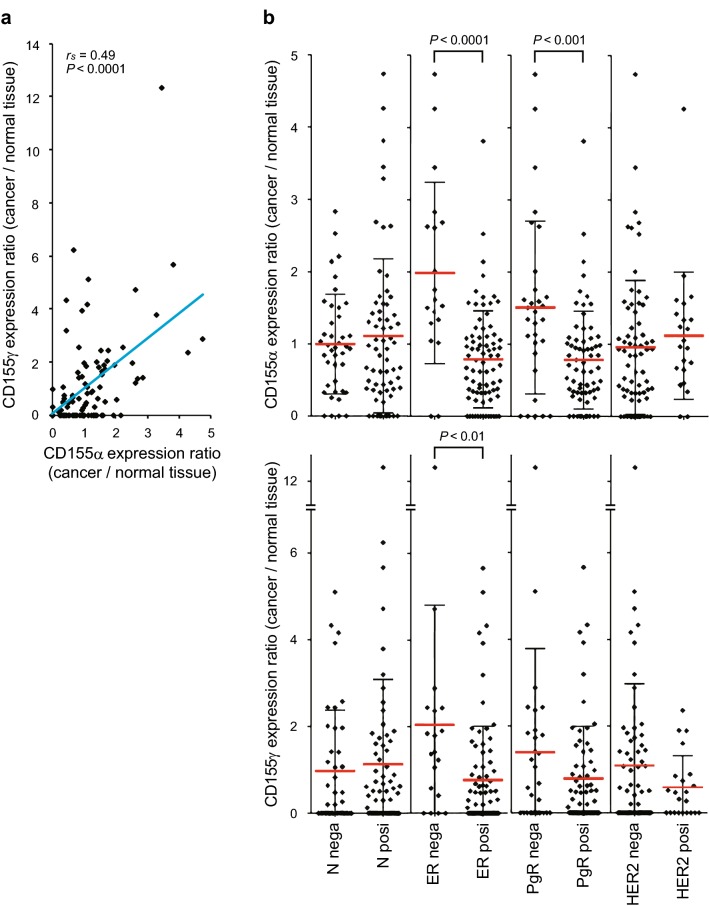
Correlation between pathological parameters and CD155 expression in breast cancers in TCGA data. **a** Correlation between expression ratios (cancer/normal) of *CD155*α and *CD155*γ. **b** Comparisons between *CD155*γ expression ratio in patients grouped by the indicated pathological parameters. *N* lymph node metastasis, *nega* negative, *posi* positive, *ER* estrogen receptor, *PgR* progesterone receptor, *HER2* human epidermal growth factor receptor 2, *r*_*s*_ Spearman rank-order correlation

Next, for *CD155*α and *CD155*γ, we calculated the ratios of mRNA levels in breast cancer tissues versus normal breast tissues of 109 patients using TCGA open access data. We found a significant positive correlation between the expression ratios of *CD155*α and *CD155*γ mRNA (Fig. 2a). The expression ratios of *CD155*α were significantly higher in ER-negative and PgR-negative breast cancers than in ER-positive and PgR-positive breast cancers, respectively (Table 2, Fig. 2b). However, the expression ratio of *CD155*γ was only significantly higher in ER-negative cancers than in ER-positive cancers (Table 2, Fig. 2b). There was no correlation between both *CD155*α and *CD155*γ expression and disease stage, and there was no significant difference between both *CD155*α and *CD155*γ expression and lymph node metastasis state (Table 2, Fig. 2b).

**Table 2:** Clinicopathological parameters and CD155 expression of breast cancers in TCGA open access data

Clinicopathological parameter	Number (%)	CD155α expression ratio				CD155γ expression ratio			
		Mean	Median	Range	Significance	Mean	Median	Range	Significance
Age (years)									
30–39	9 (8.3)	1.847	1.449	0.426–4.738	*r*_*s*_=− 0.060	1.712	0.738	0–5.661	*r*_*s*_=− 0.008
40–49	30 (27.5)	0.88	0.932	0.000–2.206	*P *=0.538	0.756	0.310	0–5.104	*P *= 0.933
50–59	25 (22.9)	1.049	1.008	0.000–3.284		0.775	0.466	0–3.789	
60–90	45 (41.3)	1.038	0.845	0.000–4.259		1.281	0.484	0–12.319	
Histological type									
Infiltrating ductal carcinoma	94 (86.2)								
Mixed	10 (9.2)								
Medullary carcinoma	2 (1.8)								
Mucinous carcinoma	1 (0.9)								
Other	2 (1.8)								
Stage									
1	19 (17.4)	0.909	0.949	0.000–2.525	*r*_*s*_= − 0.015	1.087	0.484	0–5.104	*r*_*s*_= 0.025
2	62 (56.9)	1.12	0.995	0.000–4.259	*P *= 0.89	1.037	0.291	0–12.319	*P *= 0.79
3–4	28 (25.7)	1.046	0.724	0.000–4.738		1.077	0.459	0–6.236	
Lymph node metastasis									
Negative	41 (37.6)	0.998	0.949	0.000–2.830	*P *= 0.90^a^	0.979	0.203	0–5.104	*P *= 0.73^a^
Positive	65 (59.6)	1.109	0.929	0.000–4.738		1.125	0.508	0–12.319	
Unknown	3 (2.8)								
ER status by IHC									
Negative	19 (17.4)	1.987	1.614	0.000–4.738	*P *<0.0001*^a^	2.039	1.415	0–12.319	*P *< 0.01*^a^
Positive	79 (72.5)	0.79	0.710	0.000–3.812		0.769	0.200	0–5.661	
Unknown	11 (10.1)								
PgR status by IHC									
Negative	30 (27.5)	1.509	1.396	0.000–4.738	*P *< 0.001*^a^	1.395	0.628	0–12.319	*P *=0.25^a^
Positive	67 (61.5)	0.78	0.672	0.000–3.812		0.79	0.269	0–5.661	
Unknown	12 (11.0)								
HER2 status by FISH									
Negative	68 (62.4)	0.959	0.858	0.000–4.738	*P *= 0.21^a^	1.088	0.305	0–12.319	*P *= 0.68^a^
Positive	22 (20.2)	1.121	1.012	0.000–4.259		0.586	0.390	0–2.366	
Unknown	19 (17.4)								

*Significant *P* values

^a^Mann–Whitney *U* test

*r*_*s*_ Spearman rank-order correlation, *ER* estrogen receptor, *PgR* progesterone receptor, *IHC* immunohistochemistry, *HER2* human epidermal growth factor receptor 2, *FISH* fluorescence in situ hybridization

## Discussion

Here, we showed that serum levels of sCD155 in breast cancer patients were correlated with disease stage and tumor size, suggesting that the sCD155 level in sera depends on tumor burden. These results are consistent with our previous report that sCD155 levels are higher in patients with advanced-stage gastric cancer than in those with early-stage disease and that sCD155 levels in sera are correlated with tumor size in a mouse model [19]. Although there was no correlation between the expression ratio of *CD155*γ in cancer tissues and the disease stage, serum levels of sCD155 and the expression ratio of *CD155*γ mRNA were higher in patients with ER-negative breast cancer than in those with ER-positive cancer. These results suggest that the serum sCD155 level reflects the tumor burden, especially in ER-negative breast cancer.

Recent studies have revealed that intrinsic subtypes of breast cancers characterized by ER-negative, PgR-negative, and HER2-negative expression (i.e., triple-negative breast cancer [TNBC]), or by ER-negative, PgR-negative, and HER2-positive expression have a poor prognosis [22–24]. In addition, high expression of Ki-67 is associated with a poor prognosis for breast cancer patients [25–27]. Although, in the current study, no prognostic evaluation was conducted owing to the short observation period and the small populations of TNBC and HER2-positive breast cancer, our observation of higher serum levels of sCD155 in patients with ER-negative and high Ki-67 breast cancers suggests that high levels of sCD155 in the serum might be useful for predicting poor prognosis in patients with breast cancer.

Although we observed that sCD155 inhibited the cytotoxic activity mediated by DNAM-1 on NK cells in vitro (unpublished observation), the functional role of sCD155 in tumor immunity in vivo remains unclear. Although the activating receptor DNAM-1 and the inhibitory receptors TIGIT (T cell immunoreceptor with Ig and immunoreceptor tyrosine-based inhibitory motif domains) and CD96 are able to bind to membrane-bound CD155 [28, 29], it remains undetermined (a) whether these receptors can also bind to sCD155 in vivo, (b) which receptor (if any) shows higher affinity to sCD155, and (c) whether sCD155 shows either antagonistic or agonistic activity to CTL and NK cells if it binds to either receptor. These questions are important to clarify the pathophysiological role of sCD155 in tumor immunity.
